# Drivers and forecasting of carbon emissions with extended LMDI and Bagging models: A case study of China’s Bohai Rim region

**DOI:** 10.1371/journal.pone.0322858

**Published:** 2025-05-27

**Authors:** Wen Yu, Jianguo Lin, Shusheng Yin

**Affiliations:** 1 Environmental Science and Engineering College, Dalian Maritime University, Dalian, China; 2 School of Economics and Humanities, Jiangsu Vocational College of Agriculture and Forestry, Zhenjiang, China; Tongji University School of Economics and Management, CHINA

## Abstract

The Bohai Rim region, one of the prominent coastal economic zones in China, has garnered considerable attention regarding its carbon emission characteristics and driving factors. This paper computes the overall carbon emissions within the Bohai Rim by utilizing economic and energy usage data spanning the years 2009to 2021. The results indicate a clear pattern in carbon emissions, which rise at first before experiencing a decline, primarily driven by substantial contributions from the industrial and transportation sectors. The three-layer extended Log Mean Divisia Index (LMDI) decomposition model examines how different driving characteristics affect carbon emissions, including energy composition, industrial framework, and the level of economic advancement. The study finds that despite regional economic growth, carbon emissions are declining overall. In the industrial sector, emissions reduce in large part by optimizing the energy mix and increasing energy efficiency. Similarly, modal shifts and energy efficiency advancements positively impact emission reduction in the transportation sector. Furthermore, trends in carbon emissions are predicted using a bagging ensemble model for the Bohai Rim region’s two municipalities and three provinces from 2024 to 2060. This study also examines the carbon emission reduction potential under high-speed, low-speed, and normal socio-economic development scenarios. These findings offer an in-depth insight into carbon emissions in the Bohai Rim of China and provide theoretical foundations and policy suggestions aimed at fostering low-carbon growth in related coastal zones.

## 1. Introduction

The stability of natural ecosystems and the sustainable growth of civilizations are seriously threatened by global warming, which has drawn a lot of attention due to its effects on increasing temperatures, melting glaciers, and frequent extreme weather events [1,2]. The rapid economic development has led to urbanization, increased living standards, and significantly higher energy consumption, resulting in greater carbon dioxide emissions and other greenhouse gases. A report from the International Panel on Climate Change (IPCC) indicates that high levels of carbon emissions play a major role in driving global warming [3]. In 2006, China surpassed the United States to become the leading emitter of carbon dioxide globally [4], and its total carbon emissions made up 64.8% of the global total from 2007 to 2012 [5]. By 2022, the emissions will have reached 10.2 billion tons, or around 27.7% of the total emissions worldwide [6]. This demonstrates China’s essential role in lowering global warming rates. In 2007, the Chinese Government established the National Committee to Address Climate Change to raise non-fossil energy percentage and declining energy intensity. In 2015, China unveiled its Intended Nationally Determined Contributions (INDCs), aiming to hit the highest level of carbon emissions around 2030 and cut carbon emission intensity by 60% to 65% from the 2005 baseline by 2030 [7].

China’s coastal regions, endowed with developed industrial systems and convenient waterways, land routes, and air transportation advantages, serve as a crucial engine for economic development. The Bohai Rim region, which comprises Liaoning, Hebei, and Shandong provinces, as well as Tianjin and Beijing municipalities, has over 40 ports. It is China’s largest industrial agglomeration and northern China’s most economically developed area. However, the combination of high population density, swift urban expansion, and fast-paced economic growth has been linked to a carbon-intensive energy structure and a high-energy industrial structure, thus increasing the amount of carbon emissions. In 2015, more than 52% of China’s carbon emissions and 43% of its overall energy consumption came from the Bohai Rim area [8].The Bohai Rim region plays a crucial role in China’s carbon emissions, and therefore its carbon reduction measures are of significant importance for achieving global emission reduction targets. According to the Intergovernmental Panel on Climate Change (IPCC), China is the largest carbon emitter in the world, and as the economic engine of the country, the emission reduction outcomes in the Bohai Rim directly influence global strategies for addressing climate change. A systematic analysis of carbon emissions and their driving factors in this region can provide valuable emission reduction insights for other industrial cities worldwide. Specifically, the experiences accumulated in promoting green development, applying emission reduction technologies, and building carbon markets in the Bohai Rim can offer practical and actionable pathways for other developing countries, especially in terms of industrial upgrading and energy transformation in high-emission areas. Therefore, a thorough analysis of the factors contributing to carbon emissions, along with their geographic and distribution patterns, will also be crucial for forecasting future trends and developing aimed at reaching carbon neutrality.

This is how the rest of the paper is organized: Section 2 provides a review of the relevant literature. In Section 3, there is a discussion of the study’s geographic focus and the sources of data utilized. Section 4 details the research methodology, including the carbon emissions calculations, the three-layer LMDI decomposition structure model, and the ensemble forecasting model. Section 5 delves into the analysis of carbon emissions outcomes and explores the various factors that influence it. Section 6 gives the conclusions drawn from the study and related recommendations.

## 2. Literature review

In response to global climate change, China’s national-level carbon emissions research has received widespread attention from scholars [9–12]. The challenge of carbon emissions at both the provincial and municipal levels play a crucial role in shaping more sophisticated and regionally suited strategies for emission reduction. This issue is pivotal for the successful execution of carbon reduction plans in China. Most carbon emission studies are specific to a province. Li et al. enabled the reclassification of energy in Shandong Province by analyzing energy flows and the responsibilities associated with consumption. They also assessed carbon emissions in the province by utilizing province-specific factors tailored to carbon output [13]. Su et al. developed the Stochastic Impacts by Regression on Population, Affluence, and Technology(STIRPAT) model, integrating it with Partial Least Squares Regression (PLSR) to analyze the elements affecting carbon emissions in Fujian Province [14]. Alternatively, studies have been conducted for all Chinese provinces; e.g., Geng et al. estimated carbon emission inventories for energy use and carbon intensity for each Chinese province using the mass balance approach, which is recommended by the Intergovernmental Panel on Climate Change (IPCC) [15]. The coastal region is a hub for economic activities, including heavy industries, port logistics, and shipping. Therefore, research on carbon emissions and the drivers that influence them has a big impact on national emissions. Zhu et al. examined how urbanization influences carbon emissions by combing through remote sensing data of evening light in Zhejiang Province using a panel data model [16]. Xu et al. investigated the evolving patterns and decoupling status of carbon emissions across various sectors in Guangdong Province, revealing its driving factors for consumption-based carbon emissions [17]. Bai et al. examined the variations and underlying factors influencing carbon emissions within the Beijing-Tianjin-Hebei urban agglomeration. Results showed that carbon emissions are on a downward trend and that the manufacturing structure and the intensity of emissions contribute to lower overall carbon emissions in the Beijing-Tianjin-Hebei [18]. To assess carbon emissions in the region of Beijing, Tianjin, and Hebei, Wang et al. created a multi-region input-output model and a multi-scenario factor analysis [19]. Han et al. performed a thorough examination of carbon emissions in the Beijing-Tianjin-Hebei area, focusing on both county and municipal levels [20]. However, few scholars have conducted comprehensive studies on the Bohai Rim’s carbon emissions in provinces and cities. Wang et al. projected the total carbon emissions for each province and city between 2020 and 2050, relying only on data related to energy use, GDP, and energy structure [8]. Song et al. explored the spatial and temporal regularities, spatial correlation rows, and spillover effects of carbon emission intensity using the Moran index and the spatial Durbin model. However, they did not consider the changes in other drivers affecting carbon emissions [21]. Research limited to a single industry is not conducive to formulating comprehensive carbon reduction policies for the entire Bohai Rim region.

Identifying the factors affecting carbon dioxide emissions is vital for projecting future carbon levels and evaluating new strategies for energy conservation and emission reduction. Among many approaches, the relationship between factor decomposition indicators and drivers has been widely used in energy and the environment. The main methods with high usage rates are the Structural Decomposition Approach (SDA), the Exponential Decomposition Approach (IDA), LMDI, and the Laspeyres Index. Ang compared different index decomposition methods and conducted analysis and research. Based on specific case studies comparing the changes in carbon dioxide output stemming from industrial energy use in Canada. He believes the LMDI method provides a perfect decomposition, with the outcomes free of any unexplained residual elements. And if there are negative or zero values in the dataset, they can be replaced with a small normal number [22]. Meanwhile, the LMDI decomposition method facilitates the simultaneous analysis of multiple factors, providing insights into each factor’s role in influencing changes in the target variable. This approach is instrumental in assessing the roles of energy composition, energy efficiency, economic framework, and various other influences that contribute to the increase in carbon emissions. Taking into account the time series and data availability requirements, LMDI is therefore used to examine the drivers influencing carbon emissions across three provinces and two cities in China’s Bohai Rim region. Ren et al. then identified the slight effects of energy mix and industrial structure on the rise in industrial carbon emissions in China through the LMDI two-layer decomposition method [23]. Although the above scholars utilized the LMDI approach to examine the determinants of carbon emissions across different regions, they failed to provide an in-depth multilayered decomposition of the key industries and their major energy sources in specific regions.

Carbon emission forecasting is crucial in formulating scientific emission reduction policies, optimizing energy structure, and achieving long-term sustainable development goals. To determine whether China can achieve its commitment to reduce carbon emissions by 2030, Niu et al. developed a forecasting model for generalized regression neural networks that was optimized using the improved Fireworks Algorithm, which predicted China’s carbon emissions intensity and total emissions between 2016 and 2040 [24]. However, conventional forecasting models typically focus on the patterns found in historical data, often neglecting to factor in new information that could influence the evolving trends. Zhou et al. introduced an innovative grey-roll mechanism centred on prioritizing new information. This approach uses an average weakened buffer operator to handle the raw data series, allowing for an analysis of the trends in China’s carbon emissions [25]. Liu et al. explored the potential for China to reach peak carbon emissions by investigating ten scenarios using STIRPAT and system dynamics modelling [26]. In addition to concentrating on the dynamics of China’s carbon emissions, detailed studies have also been conducted on particular regions. For example, Ren et al. developed a method for predicting networks with rapid learning for optimizing and improving chicken flocks. Predicting Guangdong Province’s carbon emissions between 2020 and 2060, carbon neutrality will only be achieved by 2060 when the tertiary sector has a concentration of both labour and resources [27]. Pan et al. constructed a multi-intelligence inter-period optimization model (MIOM) incorporating consumer preferences and technological inputs and accumulated information to forecast 13 industrial sectors’ carbon emission patterns in Liaoning Province between 2018 and 2030 [28]. Forecasting methods focus on specific models and lack combined model optimization.

The majority of recent studies on carbon emissions concentrate on the national average or individual provinces. However, the most economically developed coastal regions of China have relatively few studies on carbon emissions. Although most related literature adopts single-layer or two-layer LMDI decompositions, these studies usually fail to further explore key industries in detail based on overall industry analysis. Meanwhile, the accuracy of the forecasting models for small sample data is not high, and the consideration of the optimal fitness of multiple models is missing. This paper’s innovations include the following: (1) Calculated carbon emissions in the Bohai Rim region (Shandong Province, Liaoning Province, Hebei Province, Beijing Municipality, and Tianjin Municipality) from 2009 to 2021 based on the yearbook dataset and analyzed the features of the temporal and geographical variability in carbon emissions in combination with the regional policies; (2) Created a three-layer LMDI decomposition structure model in terms of five provinces, six industries, and eight energy sources, with a focus on analyzing carbon emission drivers for the transportation and industrial sectors; (3) In accordance with the ensemble model, carbon emissions are projected from 2024 to 2060, and the feasibility of reducing emissions in light of carbon neutrality target is explored by combining the timing of peak emissions with their maximum value across three distinct scenarios.

## 3. Study area and data sources

### 3.1 Study area

The Bohai Rim, situated along northern China’s Pacific coast, is the region’s prime coastal area. Centered around the Beijing-Tianjin-Hebei area, flanked by the Liaodong Peninsula to the north and the Shandong Peninsula to the south. The area of Bohai Rim is the most economically developed and resource-concentrated area in northern China, dominated by heavy industries, shipping, and transportation. According to the China Emission Accounts and Datasets (CEADs), it can be seen in 2021, 18.1% of the country’s GDP, 26.3% of its fossil energy consumption, and 25.2% of its carbon dioxide emissions [29].The energy consumption data shows the concentration of energy consumption and industrial production in the region. The economy of the region is highly dependent on heavy industries such as steel, coal, electricity, etc., which are typically the main sources of carbon emissions. Studying the carbon emissions in this region can more accurately identify high emission areas, providing a foundation for the formulation of relevant policies and the promotion of carbon reduction measures.

### 3.2 Data sources

This study calculated and analyzed emissions of carbon in the Bohai Rim area for the period from 2009 to 2021, using two parts of data: social statistics (population, gross regional product, and traffic turnover) and energy consumption data (actual use of natural gas, coal, coke, crude oil, gasoline, kerosene, diesel fuel, and fuel oil). Social statistics are acquired from provincial and municipal Statistical Yearbooks and Transportation Statistical Yearbooks [30], and energy consumption figures for different sectors are taken from the Energy Statistical Yearbook for each region [31]. The carbon emission metrics for the eight types of fuels are assessed and determined by selecting data from the 2006 IPCC Guidelines for National Greenhouse Gas Inventories, as well as the Guidelines for the Preparation of Provincial Greenhouse Gas Inventories in China [32,33].

## 4. Method

### 4.1 Calculation of carbon emissions

Currently, China’s national statistics have not officially released a specific methodology for calculating carbon dioxide emissions, and studies are relies on estimates of energy use and carbon emission coefficients. In light of the “Guidelines for the Preparation of Provincial Greenhouse Gas Inventories” published in 2011 by the National Development and Reform Commission and the “2006 IPCC Guidelines for National Greenhouse Gas Inventories”, this research quantifies the carbon output of the Bohai Rim region’s three provinces and two cities, taking into account the characteristics of energy statistics in the region. The following is the formula:


$$\begin{array}{c} \begin{array}{c} C=\sum_{k=1}^{n}E_{ij}^{k}\times F_{k} \end{array} \end{array}$$ (1)

Where *C* stands for carbon emissions, *E* refers to energy consumption converted into standard coal, *F* represents the carbon emission coefficient for energy type *k*, as shown in Table 1, *i* and j represent provinces (municipalities) and industry sectors, respectively, and *k* represents the type of energy, mainly comprising coal, crude oil, kerosene, coke, diesel, gasoline, fuel oil, and natural gas.

**Table 1:** Carbon emission coefficient of energy types.

Types of Energy Coefficient	Coal	Coke	Crude Oil	Kerosene	Diesel	Gasoline	Fuel Oil	Natural Gas
Standard Coal Equivalent Conversion Factor(kgce · kg^-1^,kgce · m^-3^)	0.7143	0.9714	1.4286	1.4714	1.4714	1.4571	1.4286	1.33
Carbon Emission Coefficient (CO_2_·kg^-1^ ,CO_2_· m^-3^)	0.7559	0.855	0.5857	0.5538	0.5714	0.5921	0.6185	0.4438

### 4.2 Three-layer LMDI model

In the LMDI decomposition model, carbon emissions in this paper is typically driven by multiple factors. The three-layer LMDI decomposition method employed in this paper conducts a three-level analysis. Compared with the single-layer and double-layer decomposition methods, it decomposes the influencing factors in a more comprehensive and detailed manner, enabling a clearer insight into which specific policy intervention measures are likely to be the most effective, and facilitating the formulation of more targeted policy measures. Specifically, in this paper, the change in carbon emissions is decomposed into the following three levels: eight types of energy sources, six sectors, and five regions. Based on Yu et al.’s study, this paper reveals the factors contributing to carbon emissions in two cities and three provinces in China’s Bohai Rim area using a three-layer extended LMDI methodology focusing on the industrial and transportation sectors [34]. The following is the basic LMDI model:


$$\begin{array}{c} C=\sum_{i=1}^{5}\sum_{j=1}^{6}\sum_{k=1}^{8}C_{ijk}=\sum_{i=1}^{5}\sum_{j=1}^{4}\sum_{k=1}^{8}C_{ijk}^{A}+\sum_{i=1}^{5}\sum_{j=5}\sum_{k=1}^{8}C_{ijk}^{B}+\sum_{i=1}^{5}\sum_{j=6}\sum_{q=1}^{3}C_{ij_{q}}^{P} \end{array}$$ (2)

This study will examine carbon emission drivers in the following three parts:

(1) Agriculture, Forestry, Animal Husbandry, and Fishery; Construction Industry; Wholesale, Retail, Accommodation, and Catering Services; Residential Life and Other Sectors

$$\begin{array}{c} \sum_{i=1}^{5}\sum_{j=1}^{4}\sum_{k=1}^{8}C_{ijk}^{A}=\sum_{i=1}^{5}\sum_{j=1}^{4}\sum_{k=1}^{8}\frac{C_{ijk}^{A}}{E_{ijk}}\times \frac{E_{ijk}}{E_{ij}}\times \frac{E_{ij}}{F_{ij}}\times \frac{F_{ij}}{F_{i}}\times \frac{F_{i}}{P_{i}}\times P_{i} \end{array}$$ (3)

$$\begin{array}{c} =\sum_{i=1}^{5}\sum_{j=1}^{4}\sum_{j=1}^{8}CI_{ijk}\times ES_{ijk}\times EM_{ij}\times DS_{ij}\times PCG_{i}\times P_{i} \end{array}$$ (4)

(2) Industry

$$\begin{array}{c} \sum_{i=1}^{5}\sum_{j=5}\sum_{k=1}^{8}C_{ijk}^{B}=\sum_{i=1}^{5}\sum_{j=5}\sum_{k=1}^{8}\frac{C_{ijk}^{B}}{E_{ijk}}\times \frac{E_{ijk}}{E_{ij}}\times \frac{E_{ij}}{F_{ij}}\times \frac{F_{ij}}{F_{i}}\times F_{i} \end{array}$$ (5)

$$\begin{array}{c} =\sum_{i=1}^{5}\sum_{j=5}\sum_{j=1}^{8}CM_{ijk}\times IES_{ijk}\times IEI_{ij}\times IDS_{i}\times F_{i} \end{array}$$ (6)

(3) Transportation, storage, and postal services

$$\begin{array}{c} \sum_{i=1}^{5}\sum_{j=6}\sum_{q=1}^{3}C_{ij_{q}}^{P}=\sum_{i=1}^{5}\sum_{j=6}\sum_{q=1}^{3}\frac{C_{ij_{q}}^{P}}{L_{ij_{q}}}\times \frac{L_{ij_{q}}}{L_{ij}}\times \frac{L_{ij}}{E_{ij}}\times \frac{E_{ij}}{F_{ij}}\times \frac{F_{ij}}{F_{i}}\times F_{i} \end{array}$$ (7)

$$\begin{array}{c} =\sum_{i=1}^{5}\sum_{j=6}\sum_{q=1}^{3}{CP}_{ij_{q}}\times {LW}_{ij_{q}}\times WET_{ij}\times WEI_{ij}\times WDS_{ij}\times F_{i} \end{array}$$ (8)

In this project, we concentrate on the absolute change in carbon emissions. The changes in each factor are calculated using LMDI additive decomposition. So, the following equation represents how different factors collectively participate in the breakdown of carbon emissions:


$$\begin{array}{c} \Delta C_{tot}=C^{t}-C^{t-1} \end{array}$$ (9)

$$\begin{array}{c} \begin{array}{c} =\Delta C_{CI}+\Delta C_{ES}+\Delta C_{EM}+\Delta C_{DS}+\Delta C_{PCG}+\Delta C_{P}+\Delta C_{CM}+\Delta C_{IES}+\Delta C_{IEI}+\Delta C_{IDS}+\Delta C_{F}\\ \;+\Delta C_{CP}+\Delta C_{LW}+\Delta C_{WET}+\Delta C_{WEI}+\Delta C_{WDS}+\Delta C_{F} \end{array} \end{array}$$ (10)

Where *C*^*t*^ and *C*^*t-1*^ denote carbon emissions for year *t* and the previous year *t*-1, respectively. Since *CI* and *CM* represent the carbon emission factors of fuels, which are a fixed value when the fuel is fully burned, and the type is guaranteed not to become, the carbon emission factor is assumed to have no effect. The remaining effects can be expressed as follows:


$$\begin{array}{c} \begin{array}{c} \Delta C_{ES}=\sum_{i=1}^{5}\sum_{j=1}^{4}\sum_{k=1}^{8}\omega_{ijk}^{A}\ln\left(\frac{ES_{ijk}^{t}}{ES_{ijk}^{0}}\right) \end{array} \end{array}$$ (11)

$$\begin{array}{c} \begin{array}{c} \Delta C_{EM}=\sum_{i=1}^{5}\sum_{j=1}^{4}\sum_{k=1}^{8}\omega_{ijk}^{A}\ln\left(\frac{EM_{ij}^{t}}{EM_{ij}^{0}}\right) \end{array} \end{array}$$ (12)

$$\begin{array}{c} \begin{array}{c} \Delta C_{DS}=\sum_{i=1}^{5}\sum_{j=1}^{4}\sum_{k=1}^{8}\omega_{ijk}^{A}\ln\left(\frac{{DS}_{ij}^{t}}{DS_{ij}^{0}}\right) \end{array} \end{array}$$ (13)

$$\begin{array}{c} \begin{array}{c} \Delta C_{PCG}=\sum_{i=1}^{5}\sum_{j=1}^{4}\sum_{k=1}^{8}\omega_{ijk}^{A}\ln\left(\frac{{PCG}_{i}^{t}}{{PCG}_{i}^{0}}\right) \end{array} \end{array}$$ (14)

$$\begin{array}{c} \begin{array}{c} \Delta C_{P}=\sum_{i=1}^{5}\sum_{j=1}^{4}\sum_{k=1}^{8}\omega_{ijk}^{A}\ln\left(\frac{P_{i}^{t}}{P_{i}^{0}}\right) \end{array} \end{array}$$ (15)

$$\begin{array}{c} \begin{array}{c} \Delta C_{IES}=\sum_{i=1}^{5}\sum_{j=5}\sum_{k=1}^{8}\omega_{ijk}^{B}\ln\left(\frac{IES_{ijk}^{t}}{IES_{ijk}^{0}}\right) \end{array} \end{array}$$ (16)

$$\begin{array}{c} \Delta C_{IDS}=\sum_{i=1}^{5}\sum_{j=5}\sum_{k=1}^{8}\omega_{ijk}^{B}\ln\left(\frac{IDS_{ij}^{t}}{IDS_{ij}^{0}}\right) \end{array}$$ (17)

$$\begin{array}{c} \begin{array}{c} \Delta C_{F}=\sum_{i=1}^{5}\sum_{j=5}\sum_{k=1}^{8}\omega_{ijk}^{B}\ln\left(\frac{F_{i}^{t}}{F_{i}^{0}}\right) \end{array} \end{array}$$ (18)

$$\begin{array}{c} \begin{array}{c} \Delta C_{CP}=\sum_{i=1}^{5}\sum_{j=6}\sum_{q=1}^{3}\omega_{{ij}_{q}}^{P}\ln\left(\frac{{CP}_{{ij}_{q}}^{t}}{{CP}_{{ij}_{q}}^{0}}\right) \end{array} \end{array}$$ (19)

$$\begin{array}{c} \begin{array}{c} \Delta C_{LW}=\sum_{i=1}^{5}\sum_{j=6}\sum_{q=1}^{3}\omega_{{ij}_{q}}^{P}\ln\left(\frac{L_{{ij}_{q}}^{t}}{L_{{ij}_{q}}^{0}}\right) \end{array} \end{array}$$ (20)

$$\begin{array}{c} \begin{array}{c} \Delta C_{WET}=\sum_{i=1}^{5}\sum_{j=6}\sum_{q=1}^{3}\omega_{{ij}_{q}}^{P}\ln\left(\frac{{WET}_{ij}^{t}}{{WET}_{ij}^{0}}\right) \end{array} \end{array}$$ (21)

$$\begin{array}{c} \begin{array}{c} \Delta C_{WEI}=\sum_{i=1}^{5}\sum_{j=6}\sum_{q=1}^{3}\omega_{{ij}_{q}}^{P}\ln\left(\frac{{WEI}_{ij}^{t}}{{WEI}_{ij}^{0}}\right) \end{array} \end{array}$$ (22)

$$\begin{array}{c} \begin{array}{c} \Delta C_{WDS}=\sum_{i=1}^{5}\sum_{j=6}\sum_{q=1}^{3}\omega_{{ij}_{q}}^{P}\ln\left(\frac{{WDS}_{ij}^{t}}{{WDS}_{ij}^{0}}\right) \end{array} \end{array}$$ (23)

$$\begin{array}{c} \begin{array}{c} \Delta C_{F}=\sum_{i=1}^{5}\sum_{j=6}\sum_{q=1}^{3}\omega_{{ij}_{q}}^{P}\ln\left(\frac{F_{i}^{t}}{F_{i}^{0}}\right) \end{array} \end{array}$$ (24)

In the above equation $\omega_{ijk}^{A}$ is the weighting factor defined as follows:


$$\begin{array}{c} \begin{array}{c} \omega_{ijk}^{A}=\left\{ \begin{array}{cc} \frac{C_{ijk}^{A(t)}-C_{ijk}^{A(t-1)}}{\ln C_{ijk}^{A(t)}-lnC_{ijk}^{A(t-1)}}, & C_{ijk}^{A(t)}\neq C_{ijk}^{A(t-1)}\\ C_{ijk}^{A(t)}, & C_{ijk}^{A(t)}=C_{ijk}^{A(t-1)}\\ 0, & {\;C}_{ijk}^{A(t)}=C_{ijk}^{A(t-1)}=0 \end{array}\right.\; \end{array} \end{array}$$ (25)

$$\begin{array}{c} \begin{array}{c} \omega_{ijk}^{B}=\left\{ \begin{array}{cc} \frac{C_{ijk}^{B(t)}-C_{ijk}^{B(t-1)}}{\ln C_{ijk}^{B(t)}-lnC_{ijk}^{B(t-1)}}, & C_{ijk}^{B(t)}\neq C_{ijk}^{B(t-1)}\\ C_{ijk}^{B(t)}, & C_{ijk}^{B(t)}=C_{ijk}^{B(t-1)}\\ 0, & {\;C}_{ijk}^{B(t)}=C_{ijk}^{B(t-1)}=0 \end{array}\right.\; \end{array} \end{array}$$ (26)

$$\begin{array}{c} \begin{array}{c} \omega_{{ij}_{q}}^{P}=\left\{ \begin{array}{cc} \frac{C_{ij_{q}}^{P(t)}-C_{{ij}_{q}}^{P(t-1)}}{\ln C_{{ij}_{q}}^{P(t)}-lnC_{{ij}_{q}}^{P(t-1)}}, & C_{ij_{q}}^{P(t)}\neq C_{{ij}_{q}}^{P(t-1)}\\ C_{{ij}_{q}}^{P(t)}, & C_{ij_{q}}^{P(t)}=C_{{ij}_{q}}^{P(t-1)}\\ 0, & C_{ij_{q}}^{P(t)}=C_{{ij}_{q}}^{P(t-1)}=0 \end{array}\right. \end{array} \end{array}$$ (27)

The definitions of the indices and parameters in equations (2) to (24) are shown in the S1 File.

### 4.3 Scenario setting

In this paper, we establish three scenarios to forecast carbon emissions across five regions of the Bohai Rim respectively and use an ensemble model to forecast the future trajectory of carbon emissions in the Bohai Rim between 2024 and 2060. The baseline scenario is defined based on the national and regional “Outline of the 14th Five-Year Plan for National Economic and Social Development and the Visionary Goals for 2035”, along with other relevant policy documents [35,36]. Maintaining the current economic situation and social development level, the median values of all variables are mainly set based on the historical trend of carbon dioxide emissions in each region.The setting of low-carbon and high-carbon scenarios refers to the research of Li et al. and Niu et al. on carbon peaking in Tianjin and Liaoning [37,38].The low-carbon situation aims to tighten the baseline scenario by 40%, thereby reinforcing the execution of development strategies focused on emission reduction. The high-carbon scenario is a 40% relaxation of the baseline scenario, allowing the regional economy and energy consumption to continue to rise. Table 2 shows the particular configurations of the prediction parameters.Across different carbon scenarios, the variation in variables varies, leading this study to tailor distinct parameters for each region according to their specific situations [39]. Based on trend analyses and national carbon emission control objectives, we assume that from 2024 to 2060, economic growth will persist, accompanied by decreasing energy intensity and energy consumption [40, 41]. Additionally, China introduced the “Universal Two-Child Policy” in 2015 with the aim of increasing the fertility rate. However, its impact on boosting the total population has not met the expectations set forth to date in 2017. Consequently, we predict a declining trend in population in the future [42].

**Table 2:** Prediction parameter setting.

Region	Population	GDP Growth Rate	Industrialization Level	Energy Consumption Intensity	Unit Turnover Carbon Emission
**Liaoning**	45 million by 2030, average annual decrease of 0.18% during the 14th Five-Year Plan(14^th^ FYP) [43]	6% annual growth during the 14^th^ FYP [44]	Industrial added value ≥ 6% [45]	By 2025, unit GDP energy consumption will decrease by 14.5% compared to 2020 [46]	By 2030, transportation carbon intensity down by 9.5% from 2020, annual decrease of -0.95% during the 14^th^ FYP [47]
**Baseline** **Scenario**	Decrease 0.2% every 5 years	Increase 0.5% every 5 years	Decrease 0.2% every 5 years	Decrease 0.2% every 5 years	Decrease 0.1% every 5 years
**Shandong**	103.1 million by 2025, 104 million by 2030 [48]	5.5% annual growth during the 14^th^ FYP [49]	Industrial added value ≥ 6% [45]	By 2025, unit GDP energy consumption will decrease by 15.5% compared to 2020 [50]	By 2030, vehicle carbon intensity down by 10%, ships by 5%, annual decrease of -1% during the 14^th^ FYP [50]
**Baseline** **Scenario**	Decrease 0.06% every 5 years	Increase 0.1% every 5 years	Decrease 0.2% every 5 years	Decrease 0.2% every 5 years	Decrease 0.2% every 5 years
**Hebei**	79.1 million by 2035 [51]	6% annual growth during the 14^th^ FYP [52]	Industrial added value ≥ 6% [45]	By 2025, unit GDP energy consumption will decrease by 15% [52]	By 2030, decrease by 9.5% compared to 2020, with an annual decrease rate of -0.95% [53]
**Baseline** **Scenario**	Decrease 0.05% every 5 years.	Increase 0.2% every 5 years	Decrease 0.1% every 5 years	Decrease 0.1% every 5 years	Decrease 0.1% every 5 years
**Beijing**	less than or equal 10.85 million by 2035 [54]	5% annual growth during the 14^th^ FYP [55]	Industrial added value ≥ 6% [45]	By 2025, unit GDP energy consumption will decrease by 14% [56]	By 2025, reduce by 10% from 2020, with an annual decline of -2% during the 14^th^ FYP [56]
**Baseline** **Scenario**	No population growth	Increase 0.2% every 5 years	Decrease 0.1% every 5 years	Decrease 0.2% every 5 years	Decrease 0.2% every 5 years
**Tianjin**	15 million by 2025 [57]	6% annual growth during the 14^th^ FYP [58]	Industrial added value ≥ 6% [45]	By 2025, decrease by 15% [58]	By 2030 decrease by 9.5%, annual decrease of -1% during the 14^th^ FYP [59]
**Baseline** **Scenario**	Decrease 0.24% every 5 years	Increase 0.1% every 5 years	Decrease 0.1% every 5 years	Decrease 0.1% every 5 years	Decrease 0.2% every 5 years

^a^The 14th Five-Year Plan refers to the period from 2021 to 2025.

### 4.4 Carbon emission prediction model based on integrated algorithm

Considering that the prediction of carbon emissions in the Bohai Rim has the characteristics of small samples and relatively insufficient data, to achieve accurate prediction of carbon emissions, the advantages of multiple models are integrated. This paper constructs a prediction model integrating XGBoost, SVR, RF, multiple linear regression and Lasso integrated learning. Because of multiple linear regression, lasso model is relatively traditional, so this part does not repeat. Other parts of the model are introduced as follows:

(1) XGBoost

The extreme gradient boosting regression tree algorithm (eXtreme Gradient Boosting, XGBoost) is a machine learning algorithm based on the gradient boosting framework. It has the characteristics of strong robustness, easy interpretation of data and scalability, and has good prediction ability for small sample data [60]. The basic principle is as follows:


$$\begin{array}{c} \begin{array}{c} {\hat{y}}_{i}=\sum_{k=1}^{K}f_{k}\left(x_{i}\right),f_{k}\in \varphi \end{array} \end{array}$$ (28)

where: ${\hat{y}}_{i}\;$ is for the predicted value, $x_{i}$ is the sample input, φ  is the collection of all possible decision trees, k is the number of trees, $f_{k}\;$ is the prediction result of the first tree. Therefore, the objective function can be expressed as:


$$\begin{array}{c} \varphi =\sum_{i=1}^{n}l\left({\hat{y}}_{i},y_{i}\right)+\sum_{t=1}^{K}\Omega \left(f_{k}\right) \end{array}$$ (29)

The first term is the loss function, which represents the gap between the predicted value and the actual value, and the second term is the regularization term, which is used to avoid overfitting.


$$\begin{array}{c} \Omega \left(f_{k}\right)=\gamma T+\frac{1}{2}\lambda \sum_{j=1}^{T}w_{j}^{2} \end{array}$$ (30)

Where  T represents the number of leaf nodes, w  represents the weight of leaf nodes, γ and λ algorithms are pre-defined hyperparameters, which is used to control the number and proportion of nodes, respectively.

The objective function is minimized by incremental training, added in the t-th iteration, and the objective function is updated as follows:


$$\begin{array}{c} y_{\left(t\right)}=\sum_{i=1}^{n}l\left(y_{i},{\hat{y}}_{i}^{\left(t=1\right)}+f_{t}\left(x_{i}\right)\right)+\Omega \left(f(t)\right) \end{array}$$ (31)

(2) SVR

Support vector regression (SVR) model is an extended model based on support vector machine (SVM) and regression function. With its excellent generalization performance and ability to minimize structural risk, it is often applied to the processing of small sample regression problems [61]. The Bohai Rim carbon price prediction data sample is small and suitable for this model. Based on this model, the prediction function of carbon emissions can be expressed as:


$$\begin{array}{c} y_{i}^{\prime }=f\left(x_{i}^{set}\right)=w\cdot \varphi \left(x_{i}^{set}\right)+b\; \end{array}$$ (32)

Where, $\varphi (x_{i}^{set}\backslash )$ is the mapping function, w  is the weight, b  is the intercept. By introducing the Lagrange multiplier and the kernel function, the expression of the carbon emission prediction function becomes:


$$\begin{array}{c} y_{i}^{\prime }=f\left(x_{i}^{set}\right)=\sum_{i=1}^{n}\left[\left(a_{i}-a_{i}^{*}\right)\cdot k\left(x,x_{i}^{set}\right)\right]+b \end{array}$$ (33)

Where, $k(x,x_{i}^{set}\backslash )$ is the kernel function, which plays a role of nonlinear mapping between the original space and the high-dimensional feature space.

(3) RF

Random forest (RF) is composed of multiple independent decision trees. During training, each tree is constructed based on a random sample subset and a random feature subset obtained by Bootstrap sampling. When the node is split, the optimal feature is selected from the random feature subset until the preset stopping condition is satisfied, such as the number of node samples is too small or the tree reaches the upper limit of depth. When predicting the regression problem, the average value of the prediction results of all decision trees is taken as the final output. The specific process is as follows:

Step 1: Sample set construction. Bootstrap sampling is used to construct *n* independent sample sets from the original data set, and samples are allowed to be repeated during sampling.

Step 2: Regression tree training and prunin. Construct an untrimmed regression tree based on each Bootstrap sample set. When constructing, each node randomly selects *P* features from all input features (*P* is less than the total number of features *m*), and determines the best segmentation. After the construction is completed, the decision tree is pruned to prevent overfitting.

Step 3: Prediction output. After the new data is input into the model, each regression tree in the random forest is predicted separately. Finally, all the prediction results are averaged to obtain the output of the random forest.

(4) Bagging-based ensemble algorithm

Bagging Integration is a concept in machine learning that employs the same learning algorithm to train multiple models. Bagging, a method for combinatorial prediction, reduces prediction variance by randomly sampling subsets from the original data set and constructing multiple models - such as classifiers, regression models, or other machine learning algorithms -using these subsets [62]. The basic process is as follows.

Step 1: For the prediction data $x_{i}\;$, the training sample set of each learner is extracted from the overall sample by the Bootstrap method to train the learner independently. Each learner predicts the corresponding $\;{\hat{y}}_{i}\;$, and calculates the corresponding prediction error.

Step 2: Using the minimum prediction error as the objective function, the grid search is used to assign different weights to each weak learner model to form a strong learner, thereby improving the model prediction effect.

**Fig 1:**
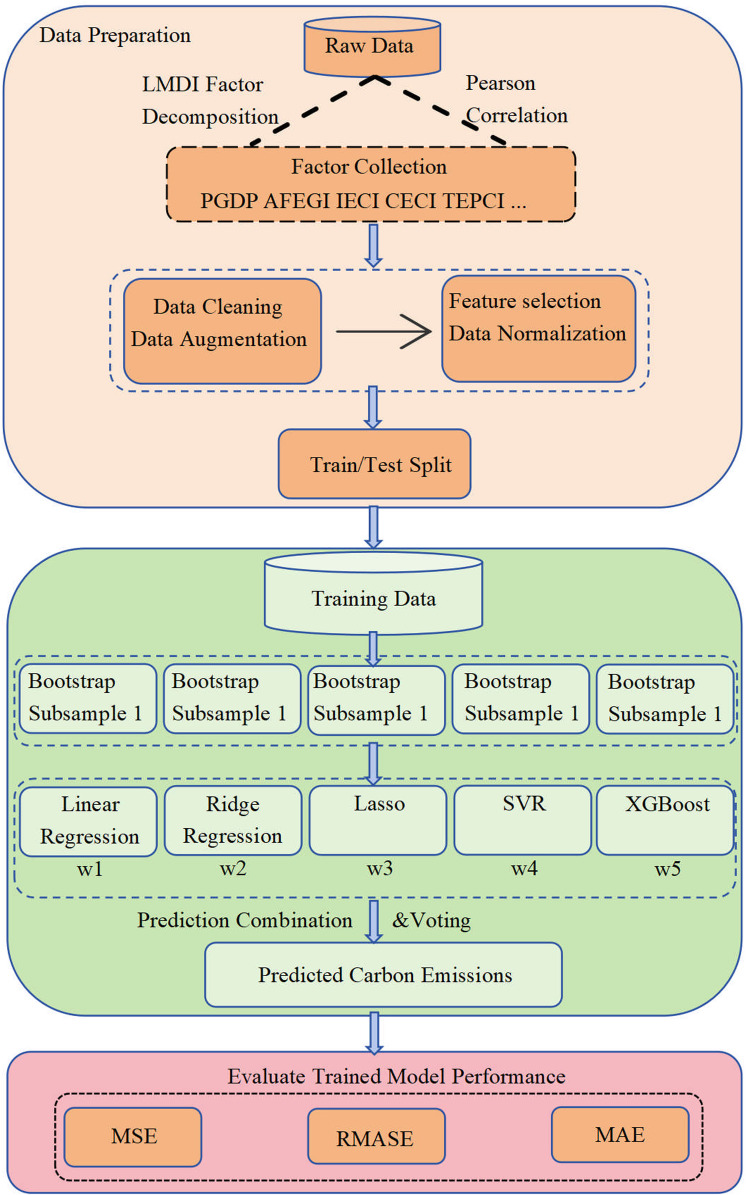
Bagging Model Algorithm Flow.

The computational framework for the Bagging algorithm applied in this paper is displayed in Fig 1.

## 5. Results

### 5.1 Carbon emission analysis

**Fig 2:**
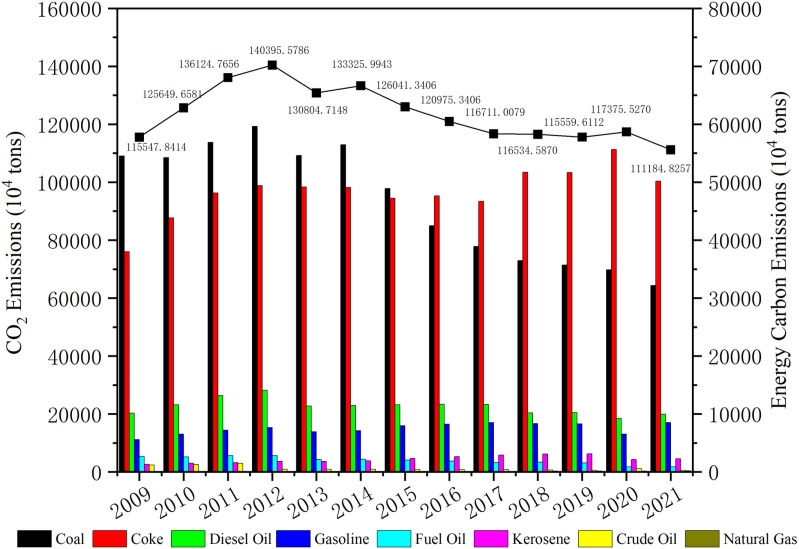
Carbon Emissions and Energy Consumption in the Bohai Rim region.

Carbon emissions in the Bohai Rim were found by calculating energy-related carbon emissions overall products for each industry. Fig 2 shows the total carbon emissions and the carbon emissions of each energy source. From 2009 (1155.48 million tons) to 2021 (1111.84 million tons), the overall trend increased at a yearly growth rate of -0.47%. As demonstrated in Fig 2, there was a dramatic rise in emissions between 2009 and 2012, culminating in a high of 1403.96 million tons in 2012. The 2008 global financial crisis prompted the Chinese Government to enact extensive economic stimulus plans, which included building high-emission, energy-intensive real estate and infrastructure. During this period, China underwent rapid industrialization and urbanization, with the Bohai Rim region, as a key industrial and economic hub in northern China, experiencing a substantial increase in energy demand, particularly for carbon-intensive energy sources like coke and coal. Between 2009 and early 2014, higher international crude oil prices forced the development of oil-related industries in the Bohai Rim, increasing diesel-related carbon emissions to 140.78 million tons. Since that time, a notable decline in carbon emissions has occurred, particularly between 2012 and 2013, when total emissions decreased by 95.94 million tons. Following this period, overall carbon emissions fluctuated slightly but remained relatively stable over time. In September 2013, China began implementing the Action Plan for Prevention and Control of Air Pollution, stepping up efforts in environmental governance and energy restructuring, aiming to improve air quality by reducing coal consumption and controlling carbon emissions from high-polluting companies [63]. It is worth noting that China’s overall policy is likely to focus more on controlling emissions from the direct combustion of coal, while the Bohai Rim region is a vital industrial and manufacturing base in China. Coke may still be a necessary consumable in some processes (e.g., steel production), which has led to a continued decrease in coal and a continued increase in coke since 2014. Also, carbon emissions from natural gas continue to rise rapidly between 2009 and 2021, rising from 410,000 tons to 1.83 million tons. Natural gas emits roughly 50% less carbon emissions per energy unit than coal. The Chinese Government has actively promoted using greener energy sources like natural gas, and the Bohai Rim region has progressively shifted from coal and other high-carbon-emitting energy sources to natural gas to reduce pollution and mitigate greenhouse gas emissions.

**Fig 3:**
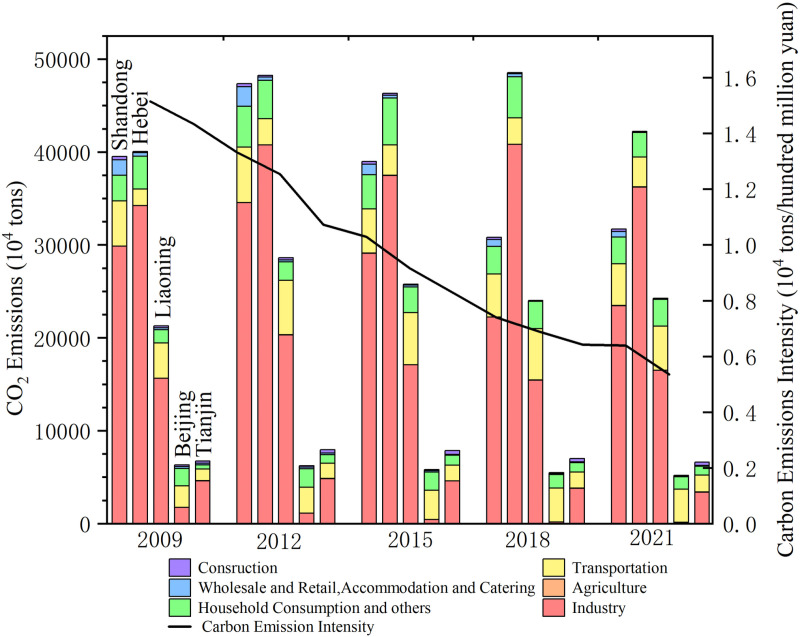
Carbon emissions and energy intensity of six sectors.

Fig 3 illustrates the differences in carbon emissions across in a variety of sectors and the relative proportions revealing the regional economic structure and energy usage. Industrial carbon emissions as a largest percentage indicates that the high energy-consuming industrial chain still dominates economic activities in China’s Bohai Rim area. In 2012, the industrial proportions in Shandong Province, Liaoning Province, Hebei Province, and Tianjin City were 83%, 72%, 68%, and 60%, respectively. Tianjin, Hebei, and Tangshan are northern China’s most extensive industrial base, known as the Beijing-Tianjin-Tangshan Industrial Base. There are more high-carbon-emitting industries in these regions, such as heavy chemical industries, iron and steel manufacturing, machinery production, and petrochemical industries, resulting in more than 50% of total industrial carbon emissions. Although the transportation industry accounts for a lesser share of carbon emissions than the industrial sector, it remains an unavoidable contributor to overall carbon output. One of the main forces behind China’s economic expansion, the Bohai Rim region has experienced rapid development of its transportation network to meet the demands of population growth and economic activities, particularly during urbanization and industrialization. This development has consequently led to a substantial increase in transportation demand. Unavoidably, this expansion has increased fuel consumption, which has raised transportation-related carbon emissions. With rising living standards in the Bohai Rim region, household energy consumption has increased, contributing to higher carbon emissions from residential energy use and daily activities. Consequently, regional carbon emissions have increased to 107.28 million tons from 104.53 million tons in 2009. However, the growth rate was not high, seeing that China’s policy push to shift its energy mix to cleaner energy sources, such as the gasification of cities and efficiency improvements in electricity use, may have partially offset the emissions impact of the rise in consumption by households. Emissions of carbon from restaurants, lodging facilities, and wholesale and retail commerce account for a smaller and gradually decreasing share of carbon emissions, with a decrease of 64.6%, but also reflect the carbon impact of the service sector in economic activity. The Bohai Rim government encourages energy savings and emission reductions by upgrading energy management systems and promoting low-carbon products. The low share of agriculture and construction in overall carbon emissions may be attributed to the following factors. First, the agricultural sector has achieved relative reductions in emissions through the adoption of modern and efficient farming methods and improved land management practices, such as agricultural intensification to enhance soil organic carbon and precision fertilizer application [64]. Secondly, the lower proportion of emissions in the construction industry may reflect the application of energy-efficient building design, innovation in building materials, and implementation of green building policies, which contribute to lowering greenhouse gas emissions and energy use. As a result, although being vital to the local economy, these two industries only make up a minor portion of the Bohai Rim’s total carbon footprint. Compared with 2009, the carbon emission intensity of the Bohai Rim in 2021 has decreased by 64.47%, and the region has realized significant emission reduction in the process of economic growth.

### 5.2 Driver analysis

**Fig 4:**
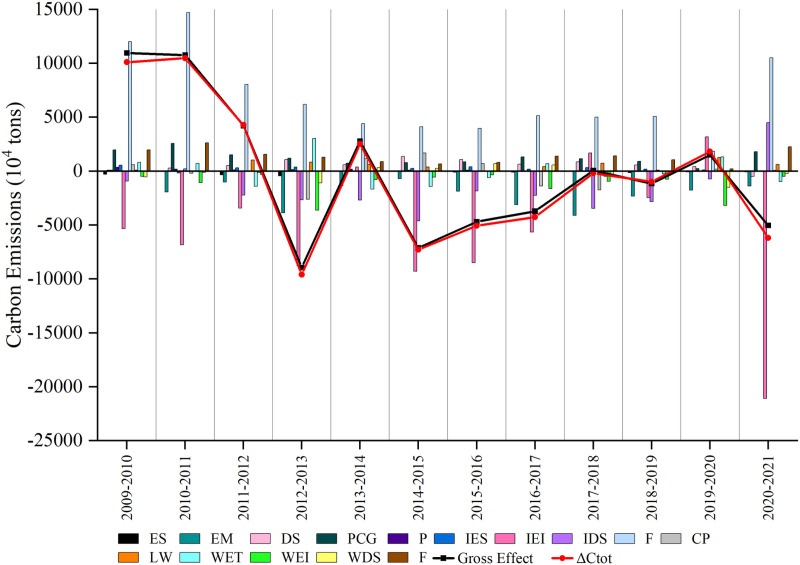
Carbon Emissions and Drivers in the Bohai Rim.

According to Fig 4, the carbon emission decomposition model shows that from 2009 to 2021, the overall impact following decomposition (black line) essentially matches the Bohai Rim region’s net change in carbon emissions trend (red line), indicating that the decomposed factors basically cover the primary motivating factors influencing variations in carbon emissions. Within the framework of the LMDI additive decomposition model, a contribution value exceeding zero implies that the factor has a positive impact on the rise of carbon emissions. Conversely, a contribution value falling below zero indicates that the factor plays a role in dampening the growth of carbon emissions. The findings indicate that between 2009 and 2021, various factors in the Bohai Rim region—such as the influence of industrial structure, economic development level, population size, energy composition in the industrial sector, level of industrialization, carbon intensity in transportation services, transportation structure, and economic level of transportation—have all positively impacted carbon emissions. In contrast, several factors in the Bohai Rim region have positively influenced carbon emissions. These include the industrial structure effect, the repercussions of economic development levels, changes in population size, the energy mix within the industrial sector, the degree of industrialization, the carbon intensity of transportation services, the transportation structure impact, and the transportation economic level effects.

#### 5.2.1. Drivers of agriculture, forestry, animal husbandry, and fishery; construction industry; wholesale, retail, accommodation, and catering services; residential life and other sectors.

**Fig 5:**
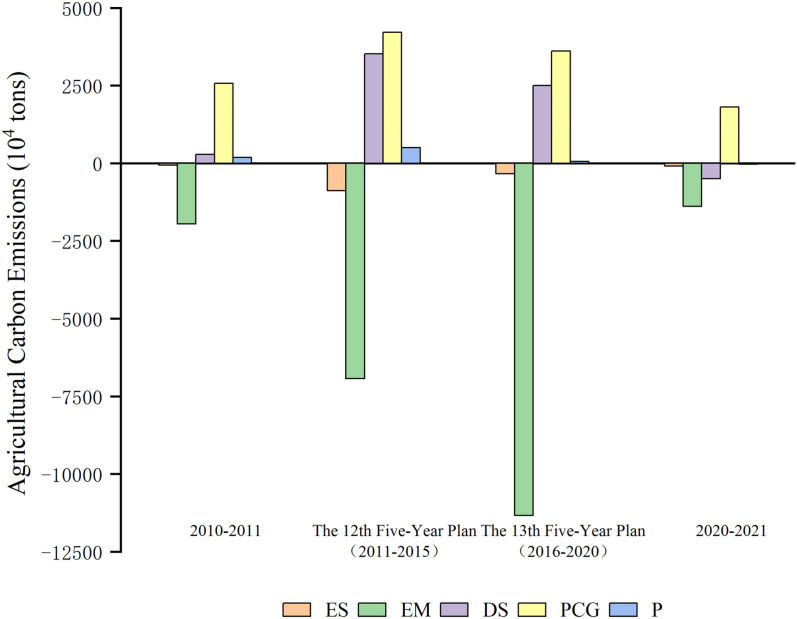
4 Periodic Carbon Emission Drivers.

These sectors form a complete economic chain from basic production to final consumption by the consumer. It reveals the level and trend of the Bohai Rim’s socioeconomic development. However, more importantly, it provides a key perspective for an in-depth understanding of environmental effects of regional economic activity as a whole. In the Bohai Rim, carbon emissions in these four sectors are mostly driven by industrial structure and economic expansion, whereas energy intensity slows the rise in carbon emissions (Fig 5).

During 2010–2011, improvements in energy efficiency, especially the application of energy-saving technologies in building construction, led to a drop in energy consumption per unit of economic production. Carbon emissions breakdown for the 12th Five-Year Plan period (2011–2015) shows significant negative values for both the energy structure effect (ES) and the energy intensity effect (EM), suggesting that these industries have improved their energy structure optimization and energy efficiency utilization [65,66]. During this period, the impact of industrial structure (DS) and economic development level (PCG) grew by 35.237 and 25.076 million tons, respectively, indicating that the total carbon emissions were under upward pressure driven by economic growth and industrial development. As economic development rises, it means that rising levels of consumption and per capita income have raised overall energy demand, which in turn raises carbon emissions.

As digital transformation accelerates, big data and smart technologies are being used to optimize energy use. The impact of energy intensity throughout the 13th Five-Year Plan (2016–2020) shows a negative value of -113 million tons, indicating improved energy efficiency. As a consequence of optimizing the economic framework, the adverse effects associated with energy structure have been reduced by 61.96%. This change is likely attributed to a deceleration in the growth of low-carbon energy consumption. The positive impact of the industry structure effect on carbon emissions has decreased, possibly because the proportion of heavy industry decreased while services and high-tech sectors, which tend to have lower energy use and carbon emissions, experienced growth during this timeframe. The growth in economic development level and the decline in the population size effect (P) suggests that the growth in carbon emissions per person has been somewhat contained while the whole economy has increased, but it still puts pressure on overall carbon emissions.

Owing to the COVID-19 that began in 2020 and continued to have an impact until 2021, the combined effect of all factors on carbon emissions declined between 2020 and 2021. Economic growth in the Bohai Rim has decelerated, affecting numerous industries, particularly the services and construction sectors. These shifts in the industrial structure have resulted in a notable decrease in total carbon emissions.

#### 5.2.2. Drivers of industry.

**Fig 6:**
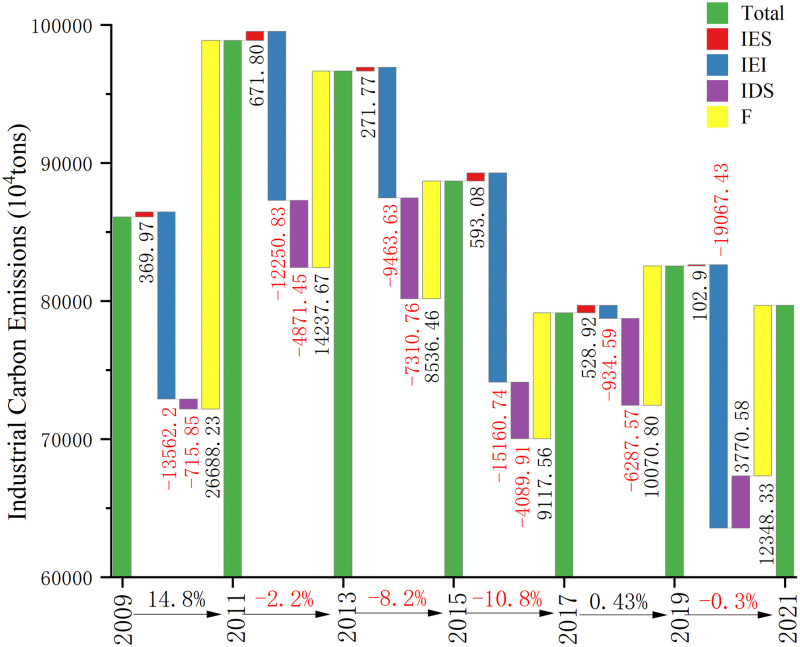
Industrial carbon emissions and driving factors.

There has been a general pattern of rising and then falling industrial carbon emissions, with a 64.068 million ton decrease overall. As shown in Fig 6 and Table 3, it increased by 14.8% from 2009 to 2011 and decreased by 2.2%, 8.2%, and 10.8% from 2011 to 2013, 2013–2015, and 2015–2017, respectively. After a slight increase (0.43%), it decreased by 0.35%. Throughout the statistical period, the industrial sector’s intensity of energy consumption (IEI) and the level of industrialization(IDS) have negatively contributed to the Bohai Rim’s whole industry’s carbon emissions. In contrast, the total economic output(F) and the structure of industrial energy consumption(IES) have significant driving effects on the industrial sector carbon emissions, with the former being more pronounced.

**Table 3:** Industrial cumulative effect and contribution.

	Cumulative effects (10^4^ tones)					Contribution ratios			
Area	$\Delta C_{IES}$	$\Delta C_{IEI}$	$\Delta C_{IDS}$	$\Delta C_{F}$	$\Delta C_{tot}$	$\frac{\Delta C_{IES}}{\Delta C_{tot}}$	$\frac{\Delta C_{IEI}}{\Delta C_{tot}}$	$\frac{\Delta C_{IDS}}{\Delta C_{tot}}$	$\frac{\Delta C_{F}}{\Delta C_{tot}}$
**Bohai Rim**	2538.43	-70439.3	-19505	80999.06	-6406.8	0.39	-10.99	-3.04	12.64
**Shandong**	898.89	-26731.75	-9374.72	28800.92	-6406.67	0.14	-4.17	-1.46	4.50
**Liaoning**	858.12	-8153.09	-4344.06	12493.72	854.69	1.01	-9.54	-5.08	14.62
**Hebei**	843.59	-29069.6	-4605.5	34821.95	1990.44	0.42	-14.6	-2.31	17.5
**Beijing**	-126.89	-2218.83	-153.51	879.06	-1620.17	-0.08	1.37	0.09	0.54
**Tianjin**	64.73	-4266.07	-1027.16	4003.41	-1225.09	0.05	-3.48	-0.84	3.27

**Fig 7:**
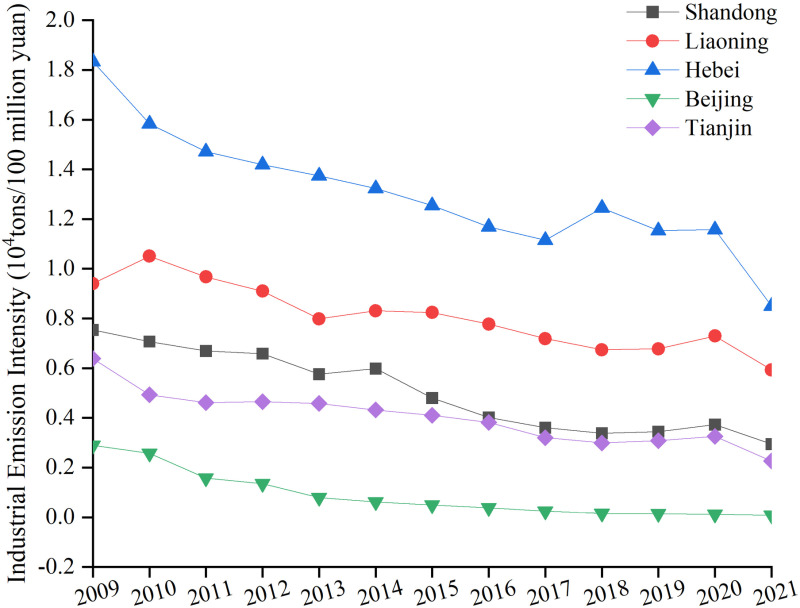
Industrial energy intensity in three provinces and two cities.

From 2009 to 2011, industrial energy intensity resulted in lower carbon emissions by 135.622 million tons. As shown in Fig 7, except for Liaoning Province, which showed a rising and then falling trend, the other four areas’ industrial energy intensity trended downward, including a 36% decrease in Hebei Province. The rise in Liaoning Province, followed by a decline, reflects the impact of technological optimization and policy adjustments after the initial industrial expansion, while the marked reduction in industrial energy intensity in Hebei is credited to ongoing enhancements in energy efficiency and the restructuring of industries with high pollution levels. Indicating that the Bohai Rim region has achieved notable advancements in improving industrial productivity and energy use efficiency. This can be attributed to the goals for reducing emissions and conserving energy established during the Eleventh Five-Year Plan, along with the revision of China’s Energy Conservation Law, which introduced strict energy efficiency and emission standards for industries with high levels of energy and emissions. These measures have significantly improved energy-use efficiency [67]. On the contrary, total economic production played a major role in the rise in carbon emissions (266.8823 million tons), as illustrated in Fig 7, with gross industrial product rising by 29.5 percentage points. Even if clean energy technology, energy structure adjustments, and emission reduction and energy-saving measures have been put into place. Although it is possible to decouple economic growth from carbon emissions, striking a short-term equilibrium between the surge in energy carbon emissions and the rapid pace of economic development proves to be a daunting task [68].

**Fig 8:**
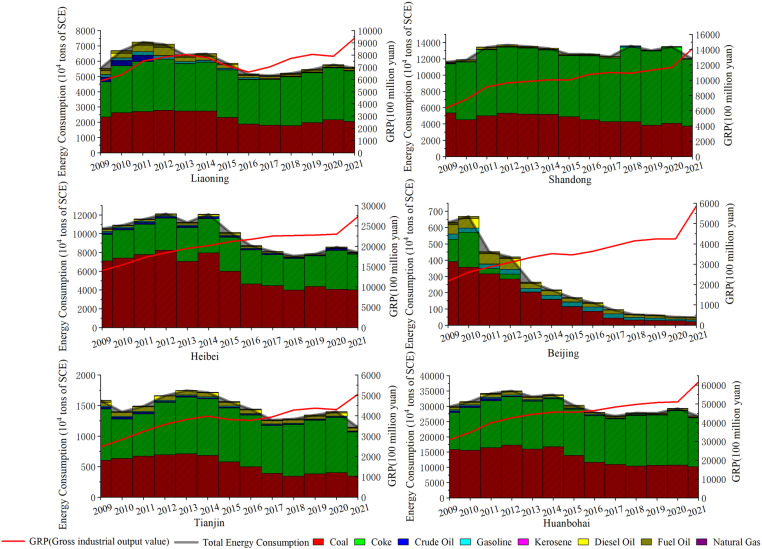
Industrial Energy Consumption and Economic Trends in the Bohai Rim and Three Provinces and Two Cities.

The slight positive growth in the structure of energy consumption in 2011–2013 (an increase of 6.718 million tons) was the result of the continued activity of energy-intensive industries, which characterizes the fact that the dependence of traditional industries on fossil fuels, such as coal, has not yet been completely lifted. As can be seen from Fig 8, coal dominates the energy consumption structure of Beijing and Hebei Province, while Liaoning Province, Shandong Province, and Tianjin Municipality use coal and coke as the main energy sources. More noteworthy is the level of industrialization that resulted in lowering carbon emissions by 41.556 million tons more than the previous period. At this time, policy guidance encourages energy conservation, emission reduction, and clean production, while improving the industry’s level of technical sophistication and promoting the transformation towards more energy-efficient and environmentally friendly production methods. Therefore, although industrial activities continue to grow, absolute reductions in carbon dioxide emissions have been achieved through improving production efficiency and adopting cleaner technologies.

The continued decline in industrial carbon emissions from 2013 to 2017 can be attributed to the synergistic effect of multiple factors, reflecting China’s macro-control and structural adjustment of its industrial development and energy consumption patterns. During this period, China initiated the 13th Five-Year Plan in 2016 after implementing the 12th [69,70].

The acceleration of industrial structure optimization has reduced the overall industrial energy intensity (246.2437 million tons of negative contribution), thereby reducing the carbon emissions per unit output. As seen in Fig 8, the Bohai Rim region has experienced an important adjustment in the structure of consumed energy, in which the percentage of coal consumption has gradually declined. Especially in Beijing, the policy orientation encourages the substitution of natural gas for coal to reduce air pollution and carbon dioxide emissions. Hebei and Liaoning provinces, as heavy industrial bases, have seen more significant reductions in energy intensity, reflecting the effectiveness of policies to eliminate outdated production capacity, implement measures to reduce emissions and save energy, and upgrade industrial technology in these regions.

Between 2017 and 2019, the influence of industrial energy intensity on carbon emissions diminished (negative contribution -93.459 million tons), indicating a slowdown in the rate of energy efficiency improvement or a relative increase in energy use intensity. However, from 2019 to 2021, this value showed a significant negative value (-1906743 million tons), partly owing to the adoption of effective energy conservation policies and advancements in industrial processes in the area. Specifically, government policy about the energy transition, including the promotion of green production technologies, incentives for energy-efficient products and services, and large-scale investments in renewable energy, have contributed to the improvement of energy use efficiency [71–73]. In addition, during this period, the transformation of traditional manufacturing industries into intelligent and service-oriented industries was actively promoted, reducing reliance on energy-intensive industries. Beijing and Tianjin are particularly focused on reducing industrial energy dependence by directing capital flows to high-tech and low-carbon industries through their policies [74,75]. Conversely, Liaoning has reduced the portion of energy-intensive industries by eliminating outdated production capacity and closing down highly polluting factories [76]. These initiatives emphasize how refining the industrial framework and advancements in technology influence energy usage and the trajectory of industrial expansion. They have made it possible to build industrial economy without using more energy, reflecting the trend toward decoupling economic development from energy consumption.

#### 5.2.3. Drivers of transportation industry.

**Fig 9:**
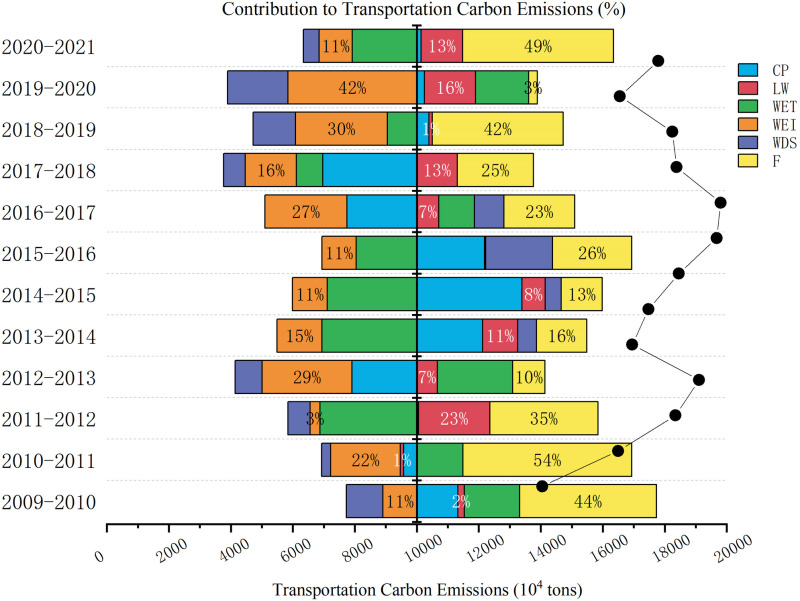
Transportation Carbon Emissions and Drivers.

The magnitude of fluctuations in sum carbon emissions from transportation in the Bohai Rim area remained relatively stable. According to Fig 9, carbon emissions experienced a substantial increase between 2009 and 2012, climbing from 140.503 million tons to 191.0747 million tons. This growth trend is intrinsically linked to the phase of swift economic expansion, the increasing demand for transportation, and the high dependence on fossil fuel modes of transportation. After peaking at 198.049 million tons in 2017, carbon emissions began to decline in 2018. This indicates that the transportation sector has achieved some success in improving energy efficiency and transportation structure throughout the 13th Five-Year Plan period. Additionally, a notable decrease in carbon emissions was seen between 2019 and 2020, particularly in 2020, when carbon emissions dropped to 165.4561 million tons. This change is most likely due to the impact of the COVID-19 epidemic, which caused a significant curtailment in the volume of transportation activities. Although carbon emissions have rebounded in 2021 due to the relaxation of transportation restrictions, they haven’t yet bounced back to their levels from before the pandemic, demonstrating the pandemic’s ongoing effects on carbon emissions from transportation.

The contribution of transportation carbon intensity (CP) exhibits large fluctuations between years. For example, between 2014 and 2015, it climbed to 33.90%, highlighting the strong driving effects of the notable rise in carbon intensity on carbon emissions from transportation. However, by 2016–2017, the contribution turned -22.50%, which hints at a reduction in carbon intensity that could be associated with energy efficiency improvements or the implementation of low-carbon measures. Transportation structure effects (LW) are not stable in contributing to carbon emissions. The contribution of LW was as high as 23.03% from 2011 to 2012, indicating that the increase in high carbon emitting modes of transportation (e.g., road and aviation) significantly contributed to carbon emissions during that period. In contrast, the contribution was negligible at 0.42% from 2015 to 2016, indicating that the transportation structure contributed very little to carbon emissions in that year. The transportation energy effect (WET) showed a positive impact in most years, especially from 2009 to 2010 when its contribution was 17.89%. However, from 2011 to 2012 and 2013–2014, the WET effect was -31.26% and -30.57%, respectively, showing that the reduction of energy use has a strong carbon emission-curbing effect. This fluctuation reveals that the efficiency and total amount of transportation energy use is a key influence on carbon emissions. The energy intensity effect (WEI) has a significant dampening impact on carbon emissions in most years. From 2019 to 2020, the contribution rate reached 41.53%, proving that enhancing energy efficiency considerably lowers carbon emissions. Similarly, the WEI effect from 2012 to 2013 was 28.99%, further proving that improving energy efficiency is an important means of controlling carbon emissions. The economic structure effect (WDS) has a comparatively smooth inhibiting impact on carbon emissions. For example, from 2019 to 2020, the contribution rate is 19.53%, showing that reorganizing the economic framework plays a role in curbing carbon emissions. This suggests that promoting a shift in the economy to low-carbon industries can effectively reduce carbon emissions from transportation. Ultimately, the transportation economic effect (F) positively affects carbon emissions in almost all years. From 2009 to 2010, the contribution was 44.07%, while by 2020–2021, it is as high as 48.64%, showing that the growth of transportation-related economic activity is a significant contributor to carbon emissions. As transportation demand increases, carbon emissions rise, indicating economic activity and carbon emissions have a comparatively substantial positive association.

### 5.3 Predictive analysis

#### 5.3.1. Prediction data preprocessing and evaluation criteria.

To deal with the input of different orders of magnitude and different dimensions, it is necessary to normalize the data. Therefore, this paper uses the formula(34) to normalize the input data:


$$\begin{array}{c} \begin{array}{c} x_{i}^{\prime }=\frac{x_{i}-\overset{―}{x}}{x_{max}-x_{min}} \end{array} \end{array}$$ (34)

Where $x_{i}^{\prime }\;$ is the normalized input, ${\;x}_{i}\;$ is the original input, $\;\overset{―}{x}\;$ is the mean, $\;x_{\max}\;$ is the maximum, $\;x_{\min}\;$ is the minimum.

To evaluate the predictive performance of the model, methods including Mean Absolute Percentage Error (MAPE), Root Mean Square Error (RMSE), and Mean Square Error (MSE) were used to test the performance of the prediction model, The calculation method is as follows:


$$\begin{array}{c} \begin{array}{c} MAPE=\frac{100\%}{n}\sum_{i=1}^{n}\left|\frac{y_{i}-{\hat{y}}_{i}}{y_{i}}\right|\; \end{array} \end{array}$$ (35)

$$\begin{array}{c} RMSE=\sqrt{\frac{1}{n}\sum_{i=1}^{n}{\left(y_{i}-{\hat{y}}_{i}\right)}^{2}} \end{array}$$ (36)

$$\begin{array}{c} MSE=\frac{1}{n}\sum_{i=1}^{n}{\left|y_{i}-{\hat{y}}_{i}\right|}^{2} \end{array}$$ (37)

where: $y_{i}\;$ is the actual value, ${\hat{y}}_{i}\;$ is the predicted value, $\overset{―}{y}\;$ is the mean value, and n  is the number of training data.

#### 5.3.2. Prediction parameter settings.

In this paper, XGBoost, SVR and RF are used to predict the carbon emissions of each province in the Bohai Rim, and the optimal weight of each year is found based on the grid search algorithm for dynamic weighting. Due to the different data input of each province, the different model combinations of each province are also different. For example, as shown in Table 4, the carbon emissions in Shandong Province fluctuate greatly, so the RF model has the best overall performance, followed by Lasso. Therefore, the weights of RF and Lasso are 0.22 and 0.78 respectively after grid search.

**Table 4:** Comparison of prediction results of Shandong benchmark model training set.

Model	MSE	RMSE	MAPE	Best parameter combination
**Bagging Integrated model(the proposed model in this paper)**	2318412.00	1362.31	0.03	Best weights: (0.0, 0.0, 0.22, 0.0, 0.0, 0.78) Best performance: 0.03557831125097361
**Multiple linear regression**	54927003.84	7411.28	0.16	–
**Ridge regression**	83679874.66	9147.67	0.22	alpha: 0.1
**Lasso**	31917464.58	5649.55	0.12	alpha: 0.001
**SVR**	25945065.12	5093.63	0.12	C:1, epsilon:0.01, gamma: auto
**XGBoost**	28995065.22	6092.33	0.15	learning_rate: 0.2, max depth: 5, n_estimators: 100
**RF**	2519442.93	1587.28	0.04	max_depth: None, min_samples_split: 2, n_estimators: 50

#### 5.3.3. Prediction results and analysis.

**Fig 10:**
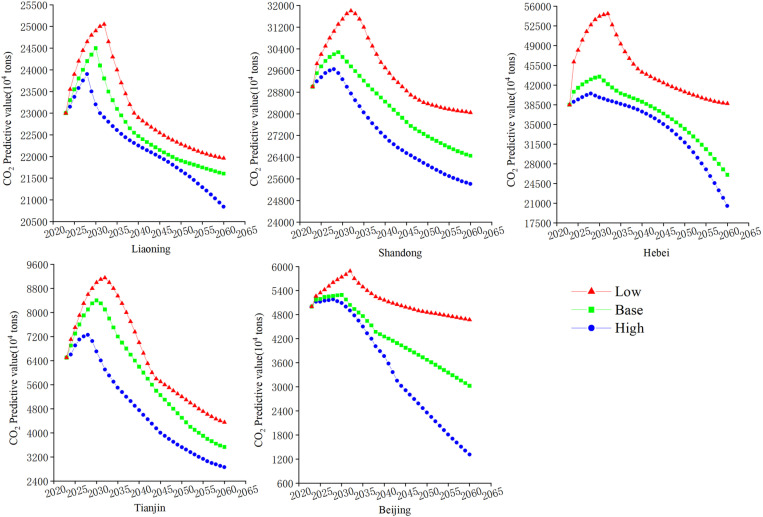
Predicted carbon emissions for three provinces and two cities in the Bohai Rim region.

Fig 10 presents the projected carbon emissions for the period from 2024 to 2060 for three provinces and two cities located in the Bohai Rim region. In the baseline projection, carbon emissions in Liaoning Province are gradually increasing from 2024 to 2030, averaging a yearly growth rate of 0.84%. This growth trend indicates that, under the current policy framework and technological conditions, economic activities and energy consumption in Liaoning Province remain dominated by the traditional high-carbon model, making it challenging to effectively curb carbon emissions in the short term. They are expected to hit their zenith in 2030, marking a 5.15% rise above the 2024 levels, resulting in a total of 245 million tons of emissions. Thereafter, it gradually decreases by 11.82% by 2060, indicating that the current policies and technologies as well as the social situation in Liaoning Province are very effective for the realization of carbon peaking and carbon neutrality. Although current policies and technologies have somewhat slowed the growth rate of carbon emissions, achieving carbon peak by 2030 and carbon neutrality by 2050 will require more aggressive policy interventions and technological innovations. In the low carbon scenario, the peak is brought forward to 2028 and the peak is 2.45% lower than the baseline scenario. By 2060, it will be reduced to 208.42 million tons, indicating that low-carbon measures have promoted the transformation of Liaoning Province’s industrial structure and effectively laid the foundation for carbon neutrality. In this scenario, the policy impact is particularly significant. By increasing investment in green technologies and clean energy, and strengthening energy efficiency and carbon capture technologies, Liaoning Province can achieve carbon peak ahead of schedule and lay a solid foundation for the carbon neutrality target.However, in the high carbon scenario, the peak is delayed until 2032 and the peak rises by 2.24% from the baseline scenario, suggesting that it will be challenging to reach carbon peaking by 2030 if left unchecked in the high carbon development scenario.Without effective policy and technological controls, carbon emissions in Liaoning Province will continue to grow in the short term and may miss the opportunity to reach carbon peak. The forecast results of the high-carbon scenario highlight the urgency of accelerating the deployment of clean energy technologies, strengthening policy guidance, and promoting the green transformation of industrial structures.

Carbon emission projections for Shandong Province show a similar trend. In the baseline scenario, carbon emissions in Shandong Province are in a slowly increasing trend between 2024 and 2029, with an average yearly increase of 0.52%, peaking early in 2029. This trend reflects the continuation of current economic growth and energy consumption patterns, especially in the context of heavy industry and heavy dependence on fossil fuels, where carbon emissions will continue to increase, albeit at a slower pace. However, compared to the baseline scenario, the predictions of the low-carbon scenario show significantly different trends. Under the low carbon scenario, the peak shifts to 2028 and 2028–2060 carbon emissions began to decline slowly, decreasing annually by an average rate of -0.48%. By 2060, emissions are projected to reach 254.1 million tons, a decline of 14.30% from the peak in 2028. This trend reflects the positive impact of low-carbon policies and measures, particularly through the optimization of industrial structure and transformation of the energy mix. Shandong Province can reduce its reliance on high-carbon energy and promote the development of clean energy. Policy measures under the low-carbon scenario, such as increasing investment in green technologies, promoting renewable energy, and improving energy efficiency, will play a key role in reducing carbon emissions and effectively advancing the carbon neutrality process. Under the high-carbon scenario, The peak is postponed until 2032, with a decline rate of 11.79% to 280.5 million tons by 2060. This suggests that Shandong Province can effectively adjust its industrial and energy structure through low-carbon measures and promote a carbon-neutral process.

The carbon emissions of Hebei Province are significantly higher than those of other provinces. Under the baseline scenario, they peaked in 2030 at 435.0 million tons and then fell by 40.11% to 260.51 million tons in 2060. The baseline scenario reflects that the current policies and technologies in Hebei Province have not fully achieved low-carbon transformation. Although carbon emissions will begin to decrease after 2030, the magnitude of this reduction is relatively slow, indicating that the current pace of carbon reduction may not meet the deadline requirements for carbon peak and carbon neutrality. After hitting its zenith, the low-carbon scenario shifts the peak to 2028, with an average annual decrease of 2.1%. By 2060, it has dropped to 205.33 million tons, a 49.30% decrease from the 2028 high. The distinctive feature of this scenario is the rapid transformation of both industrial and energy structures. The implementation of low-carbon policies has driven profound changes in energy production and consumption in Hebei Province. For example, reducing the share of high-carbon industries, increasing investment in renewable energy and clean technologies, and promoting technological innovation and energy efficiency in the industrial sector have led to a significant reduction in carbon emissions. Policy measures under the low-carbon scenario, including promoting energy structure optimization, advancing green finance, and strengthening carbon pricing mechanisms, may incur certain economic costs in the short term, but in the long run, they lay a solid foundation for achieving the carbon neutrality target. The high carbon scenario, on the other hand, is delayed until 2032, with a 25.75% increase in the peak from the baseline scenario and a 2-year delay in reaching the peak from the baseline scenario. This indicates that if Hebei Province continues to rely on traditional high carbon industries and energy without effective policy intervention and technological innovation, it will face intensified carbon emissions growth and miss the key window period for carbon peak in 2030. Carbon emissions gradually decline after peaking in 2032, falling to 38.7 million tons by 2060, a 29.25% decrease from the 2032 peak. Industrial transformation and scientific and technological innovation in Hebei Province are key factors in reaching carbon neutrality and peak carbon.

According to the baseline scenario, Tianjin’s carbon emissions are anticipated to peak at 84 million tons in 2030 before falling dramatically to 35.3 million tons by 2060, a 57.98% decrease from the 2030 high. This trend shows that, with current policies and technologies, Tianjin’s carbon emissions will be gradually curbed. However, the reduction rate will be relatively low. The baseline scenario reveals Tianjin still depends strongly on traditional high-carbon industries during economic growth. Despite gradually taking some emission-reduction steps, it has yet to achieve a full-fledged low-carbon transformation. Under the low carbon scenario, Tianjin reaches the peak in 2028 ahead of schedule and drops to 28.6 million tons by 2060, reducing emissions by 60.55% and showing strong potential for emission reduction. By accelerating industrial upgrading and energy transition, Tianjin can achieve significant emissions reductions. Under the low-carbon scenario, Tianjin not only increases investment in green technologies and clean energy but also promotes the development of advanced manufacturing and emerging industries. This complements Beijing’s strategy of relocating non-capital functions. By optimizing its industrial structure, Tianjin can effectively reduce carbon emissions and lay a solid foundation for the carbon neutrality target. The emission reduction potential in the low-carbon scenario is substantial, highlighting the importance of low-carbon policies, technological innovation, and industrial transformation. These factors can enable Tianjin to reach carbon peak and achieve carbon neutrality ahead of schedule. In the case of high carbon, the peak is reached in 2032, and the peak rises by 8.93% compared with the baseline scenario and falls to 43.5 million tons in 2060, which is 52.46% less than the peak in 2032. The slower emission reduction rate under high carbon scenarios reflects the lag in industrial restructuring and insufficient improvement in energy efficiency, which means that Tianjin will face greater challenges in achieving carbon peak by 2030 and miss the opportunity to achieve carbon neutrality goals.

The Bohai Rim’s lowest carbon emissions are found in Beijing, peaking at 52.9 million tons in 2030 under the baseline scenario, with a reduction of 42.81% by 2060. The emission reduction trend in the baseline scenario reflects the effectiveness of policy direction and technological innovation in curbing carbon emission growth while pursuing high-quality economic development and green transformation. Although carbon emissions will peak, the reduction is moderate, indicating that Beijing needs to intensify efforts to achieve its carbon peak and carbon neutrality targets. Under the low-carbon scenario, Beijing’s carbon emissions will peak ahead of schedule in 2028 and decrease by 74.57% by 2060, making it the city with the largest emission reduction in the Bohai Rim region. This scenario reflects significant progress in Beijing’s technological advancement, emerging industry development, and deep optimization of its energy structure. The substantial emission reduction potential in the low-carbon scenario demonstrates that Beijing has successfully accelerated its carbon peak by increasing investment in renewable energy, accelerating the application of clean technologies, and optimizing its industrial structure. This success not only proves the importance of technological innovation and industrial transformation but also emphasizes the key role of policies in driving the low-carbon economy. Beijing’s low-carbon path provides valuable experience for other cities and regions, especially in accelerating the carbon neutrality process through green technologies, green finance, and industrial policies. In contrast, under the high-carbon scenario, Beijing’s carbon emissions will peak in 2032, with a relatively small reduction of only 20.62%. This scenario indicates that if Beijing fails to further strengthen policy support, technological innovation, and industrial green transformation, carbon emission growth will persist to some extent. Although emissions will start to decline after 2032, the lack of more aggressive reduction measures results in a relatively low final reduction. The small reduction under the high-carbon scenario highlights the continued dominance of traditional high-carbon industries and the slow pace of energy and industrial structural transformation, making rapid emission reductions difficult.

## 6. Conclusion and policy recommendations

This paper analyzes various corporations’ carbon emissions, examines the pertinent driving forces of carbon emissions, and offers a thorough examination of the geographical and temporal patterns of carbon emissions in the Bohai Rim region. It is evident that industry and transportation are the primary contributors to regional carbon emissions, while economic growth, population change, and the configuration of industries are the key drivers behind the rise in carbon emissions. Meanwhile, the increasing trend in carbon emissions has been somewhat offset by gains in energy efficiency. This paper utilizes an advanced LMDI decomposition approach to measure the impact of various factors, including energy mix and economic growth level, on carbon emissions. This process sheds light on the respective contributions of different driving forces to the alterations in carbon emission. In addition, we use a Bagging ensemble model to predict carbon emission trends and explore the potential for lowering emissions in the context of achieving carbon neutrality goals. The research results indicate that, while the Bohai Rim region has experienced rapid growth in carbon emissions, emissions have begun to stabilize, and, with the dual impact of policy optimization and technological advancements, carbon emissions are expected to gradually decline in the future. This is closely in line with the deadline requirement of China’s carbon neutrality target (achieving carbon neutrality by 2060), which means that the carbon emission control experience in the Bohai Rim region can provide strong reference for low-carbon transformation in other regions of the country. Therefore, this study not only provides scientific support for low-carbon development in the Bohai Rim region but also offers useful insights for achieving China’s overall carbon peak and carbon neutrality targets.

Taking into account the results from the Bohai Rim’s carbon emissions study, this paper presents a range of policy suggestions designed to encourage low-carbon growth in the region. First, optimization of the energy structure ought to be the primary focus and gradually advance the development and use of sustainable and clean energy sources to lessen reliance on fossil fuels, especially in the coastal areas. This policy directly targets the carbon intensity driving factors of energy consumption. Reducing the use of coal and increasing the proportion of renewable energy will lower the carbon content per unit of energy. And take full advantage of rich natural resources, particularly the potential of offshore wind energy, to realize the green transformation of the energy structure. Additionally, promoting industrial upgrades, energy conservation, and emission reductions is crucial. This may be done by promoting the advancement of carbon-free technology and directing technological change in sectors that use energy. The policy targets both industrial energy intensity and carbon intensity drivers. By upgrading industrial technologies, energy consumption per unit of output can be reduced, while technological innovation can lower carbon emissions per unit of energy used. For the transportation industry, the encouragement of energy-efficient automobiles and the emphasis on developing low-carbon modes of transportation, such as waterways, will significantly reduce the carbon footprint of this field. This policy aims to reduce the carbon intensity and transportation turnover in the transport sector, with a focus on transitioning from high-carbon transportation modes (such as diesel vehicles and private cars) to low-carbon alternatives (such as electric vehicles and ferries). In addition, improving energy utilization efficiency can be accomplished by the widespread application of intelligent management and energy-saving technologies. Connection with driving factors: This policy reduces energy intensity by decreasing the amount of energy required for a given level of economic output or lifestyle activity. Whether it is energy efficiency management systems in industrial production or green standards in the building industry, they all contribute to improving overall energy utilization efficiency. To better motivate enterprises and individuals to participate in low-carbon development, the implementation of a carbon trading system will help promote voluntary emission reduction by enterprises through economic means, thereby promoting the application of low-carbon technologies. The carbon trading system directly targets the carbon intensity driving factor, as it increases the cost of carbon emissions and encourages the adoption of low-carbon technologies and practices. Concurrently, the improvement of public environmental awareness is also an important component of achieving low-carbon goals. This policy indirectly influences the activity level and energy intensity driving factors by incentivizing individuals to reduce energy consumption and adopt more sustainable lifestyles. Through policy incentives and publicity education, individuals are guided to reduce energy consumption in their daily lives, and practical actions are taken to support low-carbon development. In addition, regional coordinated development should be valued, and cities and provinces along the Bohai Rim need to strengthen exchanges and cooperation in clean energy technology and management experience to achieve common progress in carbon emission control. This policy addresses multiple driving factors, such as activity level, energy intensity, and carbon intensity, by promoting knowledge sharing and collaboration in clean energy technologies and efficient practices.

There are still certain issues that require improvement even though this research thoroughly examines the characteristics of carbon emissions and the variables affecting them in the Bohai Rim area. First, the limitation of data sources is an unavoidable problem in this study, especially in the breakdown and precision of energy consumption data, and the data’s unpredictability might compromise the results’ veracity. Future research could consider integrating more real-time monitoring data to enhance the data’s dependability. Second, the adopted three-level extended LMDI decomposition model and Bagging ensemble forecasting model have certain limitations in dealing with exogenous variables, especially the insufficient consideration of factors such as policy changes and market fluctuations, which might cause the forecasted outcomes to deviate from the real circumstances. Therefore, more dynamic factors should be introduced in the future to enhance the adaptability of the models. In addition, although this paper proposes industry analysis for the Bohai Rim region’s carbon emissions, the detailed analysis of some high energy-consuming industries is relatively insufficient and fails to dig deeper into the carbon emission reduction paths of specific industries. Future research should further refine the classification of industries and provide more specific emission reduction strategies so as to respond to the carbon emission problems of energy-consuming industries in a more targeted manner. Finally, the complexity and uncertainty of policy implementation also pose a challenge to the practical application of the findings, especially as local governments may face problems such as lack of funds and conflict of interest in implementing environmental policies, which to some extent affect the actual effects of the policies. Therefore, further studies should focus more on the socio-economic barriers at the policy implementation level to ensure that the proposed policy recommendations can be practically implemented in practice.

## Supporting information

S1 File The symbols and definitions of equations (2) to (25). (DOCX)

S2 Dataset The base data used for the factor decomposition analysis. (XLSX)
