# Supplementary material for: Drivers and forecasting of carbon emissions with extended LMDI and Bagging models: A case study of China’s Bohai Rim region
Source: PLoS One. 2025 May 27;20(5):e0322858. doi: 10.1371/journal.pone.0322858 (PMC12111488; doi:10.1371/journal.pone.0322858)
Supplement: S1 File — (DOCX) [file pone.0322858.s001.docx]

| Appendix 1 | |
| --- | --- |
| Symbol | **Definition** |
| *i* | Refers to the three provinces and two municipalities (***i*** = 1, 2, ..., 5), specifically Shandong, Liaoning, Hebei, Tianjin, and Beijing. |
| *j* | Represents five different sectors (***j*** = 1, 2, ..., 6), specifically agriculture, forestry, animal husbandry, and fishery; industry; construction; wholesale and retail trade; accommodation and food services; transportation, warehousing, and postal services; and residential consumption. |
| *k* | Refers to the fuel type (***k*** = 1, 2, ..., 8), specifically coal, coke, crude oil, gasoline, diesel, kerosene, fuel oil, and natural gas. |
| *q* | Represents the three types of transportation (***q*** = 1, 2, 3), specifically land, water, and air transport. |
| *C* | Total CO₂ emissions. |
| $\boldsymbol{C}_{\boldsymbol{ijk}}$ | CO₂ emissions from fuel ***k*** in sector ***j*** of province ***i***. |
| $\boldsymbol{C}_{\boldsymbol{ijk}}^{\boldsymbol{A}}$ | CO₂ emissions from fuel ***k*** in agriculture, forestry, animal husbandry, fishery, construction, wholesale and retail, accommodation and food services, and other sectors of province ***i***. |
| $\boldsymbol{C}_{\boldsymbol{ijk}}^{\boldsymbol{B}}$ | CO₂ emissions from fuel ***k*** in the industrial sector of province ***i***. |
| $\boldsymbol{C}_{\boldsymbol{i}\boldsymbol{j}_{\boldsymbol{q}}}^{\boldsymbol{P}}$ | CO₂ emissions in transportation and postal sectors of province ***i*** for transport mode ***q***. |
| $\boldsymbol{E}_{\boldsymbol{ijk}}$ | Consumption of fuel ***k*** in sector ***j*** of province ***i***. |
| $\boldsymbol{E}_{\boldsymbol{ij}}$ | Total energy consumption in sector ***j*** of province ***i***. |
| $\boldsymbol{F}_{\boldsymbol{ij}}$ | The economic output of sector ***j*** in province ***i***. |
| $\boldsymbol{F}_{\boldsymbol{i}}$ | The total economic output of province ***i***. |
| $\boldsymbol{P}_{\boldsymbol{i}}$ | Total population of province ***i***. |
| $\boldsymbol{L}_{\boldsymbol{i}\boldsymbol{j}_{\boldsymbol{q}}}$ | Conversion turnover for transport mode ***q*** in the transportation sector of province ***i***. |
| $\boldsymbol{L}_{\boldsymbol{ij}}$ | Total conversion turnover for the transportation sector of province ***i***. |
| $\boldsymbol{C}\boldsymbol{I}_{\boldsymbol{ijk}}$ | CO₂ emission intensity for fuel type ***k*** in sector ***j*** of province ***i***. |
| $\boldsymbol{E}\boldsymbol{S}_{\boldsymbol{ijk}}$ | Energy consumption structure for fuel type ***k*** in sector ***j*** of province ***i***. |
| $\boldsymbol{E}\boldsymbol{M}_{\boldsymbol{ij}}$ | Energy consumption intensity for sector ***j*** in province ***i***. |
| $\boldsymbol{D}\boldsymbol{S}_{\boldsymbol{ij}}$ | Structural share of sector ***j*** in province ***i***. |
| $\boldsymbol{PC}\boldsymbol{G}_{\boldsymbol{i}}$ | Economic output share of province ***i***. |
| $\boldsymbol{C}\boldsymbol{M}_{\boldsymbol{ijk}}$ | CO₂ emission intensity for fuel ***k*** in the industrial sector of province ***i***. |
| $\boldsymbol{IE}\boldsymbol{S}_{\boldsymbol{ijk}}$ | Energy consumption structure for fuel ***k*** in the industrial sector of province ***i***. |
| $\boldsymbol{IE}\boldsymbol{I}_{\boldsymbol{ij}}$ | Energy intensity in the industrial sector of province ***i***. |
| $\boldsymbol{ID}\boldsymbol{S}_{\boldsymbol{i}}$ | Industrialization level of province ***i***. |
| $\boldsymbol{CP}_{\boldsymbol{i}\boldsymbol{j}_{\boldsymbol{q}}}$ | Carbon density per unit turnover in the transportation sector of province ***i*** for mode ***q***. |
| $\boldsymbol{LW}_{\boldsymbol{i}\boldsymbol{j}_{\boldsymbol{q}}}$ | Transport structure in the transportation sector of province ***i*** for mode ***q***. |
| $\boldsymbol{WE}\boldsymbol{T}_{\boldsymbol{ij}}$ | Energy consumption per unit turnover in the transportation sector of province ***i***. |
| $\boldsymbol{WE}\boldsymbol{I}_{\boldsymbol{ij}}$ | Energy intensity in the transportation sector of province ***i***. |
| $\boldsymbol{WD}\boldsymbol{S}_{\boldsymbol{ij}}$ | Share of economic added value in transportation sector of province ***i*** relative to total GDP of province ***i***. |
| $\boldsymbol{\Delta}\boldsymbol{C}_{\boldsymbol{CI}}$ | Carbon emission coefficient effect. |
| $\boldsymbol{\Delta}\boldsymbol{C}_{\boldsymbol{ES}}$ | Energy structure effect. |
| $\boldsymbol{\Delta}\boldsymbol{C}_{\boldsymbol{EM}}$ | Energy intensity effect. |
| $\boldsymbol{\Delta}\boldsymbol{C}_{\boldsymbol{DS}}$ | Industrial structure effect. |
| $\boldsymbol{\Delta}\boldsymbol{C}_{\boldsymbol{PCG}}$ | Economic development level effect. |
| $\boldsymbol{\Delta}\boldsymbol{C}_{\boldsymbol{P}}$ | Population scale effect. |
| $\boldsymbol{\Delta}\boldsymbol{C}_{\boldsymbol{CM}}$ | Industrial sector carbon emission coefficient effect. |
| $\boldsymbol{\Delta}\boldsymbol{C}_{\boldsymbol{IES}}$ | Industrial sector energy structure effect. |
| $\boldsymbol{\Delta}\boldsymbol{C}_{\boldsymbol{IEI}}$ | Industrial sector energy intensity effect. |
| $\boldsymbol{\Delta}\boldsymbol{C}_{\boldsymbol{IDS}}$ | Industrial sector industrialization level effect. |
| $\boldsymbol{\Delta}\boldsymbol{C}_{\boldsymbol{CP}}$ | Carbon density effect in transportation services. |
| $\boldsymbol{\Delta}\boldsymbol{C}_{\boldsymbol{LW}}$ | Transportation structure effect. |
| $\boldsymbol{\Delta}\boldsymbol{C}_{\boldsymbol{WET}}$ | Transportation energy efficiency effect. |
| $\boldsymbol{\Delta}\boldsymbol{C}_{\boldsymbol{WEI}}$ | Transportation energy intensity effect. |
| $\boldsymbol{\Delta}\boldsymbol{C}_{\boldsymbol{WDS}}$ | Transportation economic structure effect. |
| $\boldsymbol{\Delta}\boldsymbol{C}_{\boldsymbol{F}}$ | Economic output effect. |
